# Biomimetic Adaptive Pure Pursuit Control for Robot Path Tracking Inspired by Natural Motion Constraints

**DOI:** 10.3390/biomimetics9010041

**Published:** 2024-01-09

**Authors:** Suna Zhao, Guangxin Zhao, Yan He, Zhihua Diao, Zhendong He, Yingxue Cui, Liying Jiang, Yongpeng Shen, Chao Cheng

**Affiliations:** 1 College of Electrical and Information Engineering, Zhengzhou University of Light Industry, Zhengzhou 450000, China; snzhao1221@zzuli.edu.cn (S.Z.); 332101050067@email.zzuli.edu.cn (G.Z.); heyan@zzuli.edu.cn (Y.H.); 2010018@email.zzuli.edu.cn (Z.D.); hezhendong@email.zzuli.edu.cn (Z.H.); 332101030024@email.zzuli.edu.cn (Y.C.); jiangliying@zzuli.edu.cn (L.J.); 2 Key Laboratory of Bionic Engineering, Ministry of Education, Jilin University, Changchun 130022, China

**Keywords:** human–computer interaction, FWDDR, path following, bionic motion, kinematic inspiration, adaptive techniques, quadratic polynomial integration

## Abstract

The essence of biomimetics in human–computer interaction (HCI) is the inspiration derived from natural systems to drive innovations in modern-day technologies. With this in mind, this paper introduces a biomimetic adaptive pure pursuit (A-PP) algorithm tailored for the four-wheel differential drive robot (FWDDR). Drawing inspiration from the intricate natural motions subjected to constraints, the FWDDR’s kinematic model mirrors non-holonomic constraints found in biological entities. Recognizing the limitations of traditional pure pursuit (PP) algorithms, which often mimic a static behavioral approach, our proposed A-PP algorithm infuses adaptive techniques observed in nature. Integrated with a quadratic polynomial, this algorithm introduces adaptability in both lateral and longitudinal dimensions. Experimental validations demonstrate that our biomimetically inspired A-PP approach achieves superior path-following accuracy, mirroring the efficiency and fluidity seen in natural organisms.

## 1. Introduction

In recent decades, wheeled mobile robots have garnered increased attention from researchers. Owing to their exceptional load-bearing capacity, flexibility, and strong stability [1], these robots are extensively used in fields such as modern industry, logistics, transportation, and firefighting. Numerous scholars have conducted relevant research on mobile robots, making significant contributions to automatic navigation [2,3,4,5,6,7,8,9,10]. Path tracking, a key technology in automatic navigation, critically determines the performance accuracy of autonomous driving. Therefore, it is vital to study path-tracking control algorithms to enhance the autonomous navigation capabilities of robots [11].

Path tracking involves controlling the robot to follow a predetermined path closely, minimize tracking errors, and maintain high robustness against disturbances. The design of the controller is crucial for path following. Consequently, many scholars have conducted corresponding research and proposed various control algorithms. These control algorithms can be mainly categorized into direct feedback control, dynamic model-based control, and geometric tracking control. The direct feedback control primarily adjusts the wheel’s rotation angle based on system feedback without considering the robot’s model, parameters, or other information. For example, methods like the PID (Proportional Integral Derivative) control [12] and the Fuzzy control [13] fall under this category. In [14], a genetic algorithm (GA) was introduced to enhance PID tracking, effectively overcoming external interferences. In [15], fuzzy logic control was integrated with predictive control to improve the robustness and immunity of the controller and to overcome the time delay caused by the sensors. In addition, in [16,17], the hybrid algorithm of fuzzy logic and Adaptive Neuro-Fuzzy Inference System (ANFIS) also provides some inspiration. The dynamics model-based control primarily analyzes the relationship between the robot’s forces and dynamics parameters, like velocity, using the established robot dynamics model. For example, methods like Model Predictive Control (MPC) [18] and Linear Quadratic Regulator (LQR) control [19] fall under this category. In [20], an MPC-based interval trajectory tracking control method was designed to enhance the speed and stability of AGV travel in a feasible area by increasing the limit of the yaw angle. In [21], a fast rolling optimization algorithm was designed based on the LQR lateral controller for a tracked mobile robot, and the weight coefficient matrices Q and R were optimized to improve the tracking accuracy and dynamic performance of the controller. The geometric tracking control method emphasizes analyzing the robot’s geometric relationship with the reference path to determine wheel rotation angles. There are many typical representatives of geometric tracking control methods, such as the PP control method [22] and the Stanley control method. In [23], an improved PP control algorithm was proposed, which adaptively modified the pre-sight distance by selecting the temporary pre-sight point, and improved robot tracking accuracy. In [24], an adaptive steering controller based on the Stanley tracking controller was studied. This system integrates a database driven by the particle swarm optimization algorithm and a fuzzy monitoring strategy, enhancing the adaptability of armored vehicles to trajectory alterations.

Among the prevalent control methods, PID and LQR demonstrate reduced robustness against external disturbances and are less stable. MPC, though sophisticated, demands rigorous modeling and intense computational efforts, compromising its real-time efficiency. Meanwhile, the Stanley method necessitates a curvature-continuous path. In contrast, PP control offers robust resilience to external interference without specific path prerequisites. Although the algorithm simulates human driving habits and is progressive, how to determine the best forward-looking distance is still a problem.Addressing this, this paper introduces an adaptive pure pursuit (A-PP) algorithm. This algorithm enhances the quadratic polynomial in both lateral and longitudinal dimensions to adaptively adjust the forward-looking distance. This makes the tracking more accurate.

The main contributions of this paper are as follows: (1) At the lateral level, the lateral error is taken into account. When the error increases, reduce the forward-looking distance in order to eliminate the error as soon as possible and improve the response speed. When the error decreases, increase the forward-looking distance appropriately to avoid the system response overshoot. (2) At the longitudinal level, the path curvature is taken into account and the forward-looking distance is adjusted according to the road conditions of the reference path, featuring online adaption of the control parameters, especially the lateral error during the turning section.

The remaining sections of this paper are organized as follows: In Section 2, the FWDDR kinematic model is established, and the relationship between pose and velocities is elucidated by analyzing the motion characteristics of FWDDR. In Section 3, the principles of the traditional algorithm and A-PP algorithm are introduced. In Section 4, the feasibility of the A-PP algorithm is assessed via simulation. In Section 5, the A-PP algorithm’s efficacy is demonstrated in a real-world setting using FWDDR. Section 6 offers a comprehensive summary.

## 2. Kinematic Model

### 2.1. Kinematic Analysis

Generally, differential drive robots lack enough torque to drive the wheels directly. In order to augment the torque, robot wheels are often connected directly to the reduction motor. The four-wheel differential structure derives its steering power from the left and right motor differential. Power, once generated from the motors, is transmitted to both the left and right front and rear axles via a reducer, eventually reaching the wheels. The FWDDR relies on motors to drive the wheel movement independently, which has the advantages of potent driving force and agile control. During operation, deviations exist between the robot’s actual and ideal motion states. Considering the non-holonomic constraints of the FWDDR, the following conditions should be assumed [25,26,27].

(1) Four wheels of the FWDDR are symmetrically distributed on the same plane;
(2) None of the FWDDR’s wheels idle;
(3) The FWDDR does not exhibit longitudinal skidding during steering;
(4) The FWDDR possesses an ample turning radius;
(5) The FWDDR’s center of mass is situated on the robot’s x-axis.

**Figure 1:**
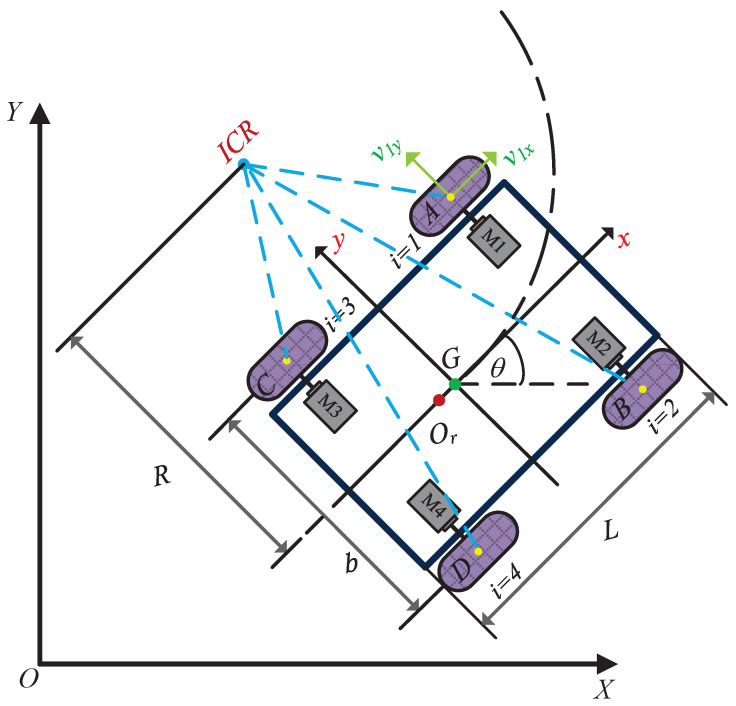
Kinematics of an FWDDR.

The kinematic diagram of the FWDDR is shown in Figure 1. XOY represents the global coordinate system, with $xo_{r}y$ illustrating the local or body coordinate system. The parameters *i* (1, 2, 3, 4) symbolize the robot’s four wheels. Points (A,B,C,D) indicate where the tire contacts the ground, *b* denotes the spacing between rear wheels, and Or and *G* symbolize the FWDDR’s particle and geometric centers, respectively.

The velocity direction of ideal contact points A,B,C, and *D* between the tire and the ground is perpendicular to the radial direction of the radius of gyration. Furthermore, it can be decomposed into longitudinal component velocity along the rolling direction of the wheel and transverse component velocity along the axial direction of the motor, which can be written as:

(1) $$\begin{array}{c} \begin{array}{c} \left\{\begin{array}{c} v_{l}=v_{1x}=v_{3x}\\ v_{r}=v_{2x}=v_{4x}\\ v_{f}=v_{1y}=v_{2y}\\ v_{b}=v_{3y}=v_{4y} \end{array}\right. \end{array} \end{array}$$

where $v_{l}$ and $v_{r}$ are the longitudinal partial velocities of the left and right wheels, and $v_{b}$ and $v_{f}$ are the transverse partial velocities of the front and rear wheels, respectively. From (1), it can be seen that the longitudinal partial velocity of wheels on the same side is equal, and similarly, the transverse partial velocity of wheels on the same end is equal [28].

### 2.2. Equation of Motion

In Figure 1, the point ICR signifies the robot’s instantaneous center of rotation, located on the *y*-axis of the center-of-mass coordinate system. Meanwhile, *R* denotes the instantaneous radius of rotation during motion [29,30].

Due to the v=ω·R, if ω is constant, *v* is proportional to *R*, the rotation angular velocity of the robot can be expressed as:

(2) $$\begin{array}{c} \omega =\frac{v}{R}=\frac{v_{r}}{R+b/2}=\frac{v_{l}}{R-b/2} \end{array}$$

where $v_{r}$ and $v_{l}$ denote the speeds of the right and left wheels, respectively, *v* signifies the linear velocity, and *w* is the rotational angular velocity.

According to (2), the following can be obtained:

(3) $$\begin{array}{c} \omega =\frac{v_{r}-v_{l}}{b} \end{array}$$

(4) $$\begin{array}{c} v=\omega R=\frac{v_{r}+v_{l}}{2} \end{array}$$

Combined with Formulas (3) and (4), the FWDDR’s instantaneous radius of rotation *R* can be expressed as:

(5) $$\begin{array}{c} R=\frac{b}{2}\frac{v_{r}+v_{l}}{v_{r}-v_{l}} \end{array}$$

Combined with Figure 1, matrix form of the kinematic model can be derived as follows:

(6) $$\left[\begin{array}{c} \overset{\cdot }{x}\;\\ \overset{\cdot }{y}\;\\ \overset{\cdot }{\theta }\; \end{array}\right]=\left[\begin{array}{cc} \cos\theta & 0\\ \sin\theta & 0\\ 0 & 1 \end{array}\right]\left[\begin{array}{c} v\\ w \end{array}\right]$$

By substituting (3) and (4) into (6), we obtain:

(7) $$\left[\begin{array}{c} \overset{\cdot }{x}\;\\ \overset{\cdot }{y}\;\\ \overset{\cdot }{\theta }\; \end{array}\right]=\left[\begin{array}{cc} \frac{\cos\theta }{2} & \frac{\cos\theta }{2}\\ \frac{\sin\theta }{2} & \frac{\sin\theta }{2}\\ \frac{1}{b} & -\frac{1}{b} \end{array}\right]\left[\begin{array}{c} v_{r}\\ v_{l} \end{array}\right]$$

Equation (7) is the general form of the kinematic model of the FWDDR, establishing the relationship between the robot’s position and the speeds of the left and right wheels.

## 3. Path-Tracking Controller Design

**Figure 2:**
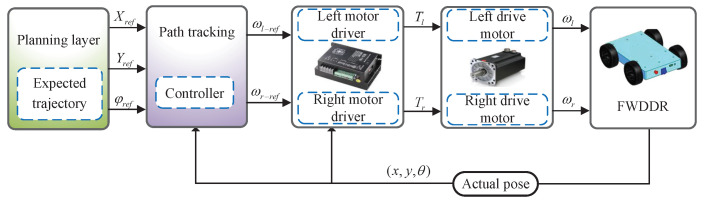
General block diagram of FWDDR path tracking.

Path tracking involves adjusting a robot’s steering to align with a desired trajectory by assessing the deviation between its current position and the trajectory’s target point. Figure 2 presents the general block diagram for FWDDR path tracking.

Reference trajectory is obtained through the planning layer. The reference trajectory’s desired attitude is relayed to the subsequent layer as the target for trajectory tracking. The subsequent layer consists of a trajectory-tracking controller, designed based on the kinematic model. It utilizes an optimization algorithm to transmit speed instructions to the motors, achieving trajectory tracking.

### 3.1. Traditional PP Control

**Figure 3:**
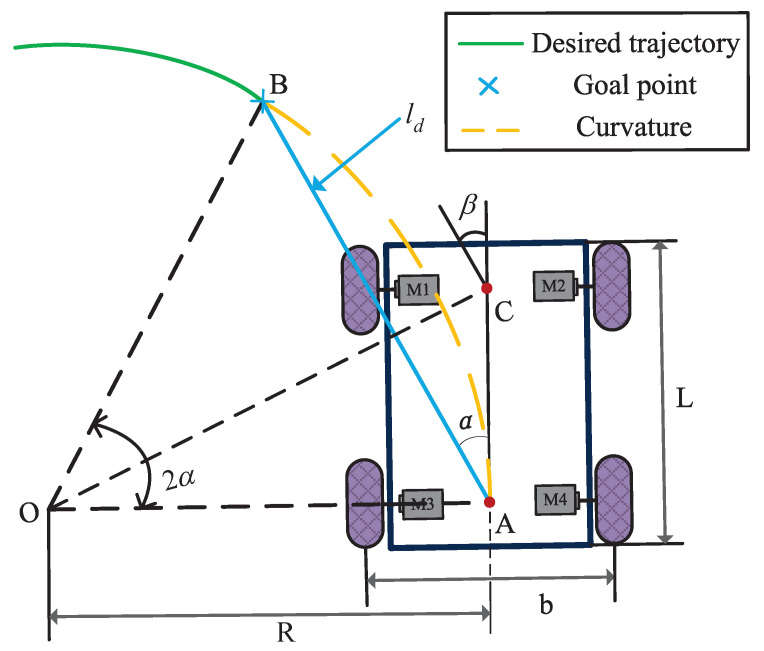
Schematic diagram of PP algorithm of the FWDDR.

The PP algorithm’s primary characteristic is its robustness against external interference, with modest path requirements. it is apt for both continuous and discontinuous path scenarios, boasting a broad application spectrum. This algorithm is a typical lateral control method. The main idea involves identifying a preview point from the rear wheel’s central position towards the desired trajectory. Then, it is assumed that the vehicle can travel along an arc passing through the preview point, and the vehicle angle is determined according to the geometric relationship among the preview distance, rotation radius, and rotation angle [31]. Thus, the mobile robot achieves precise tracking of the desired trajectory. As shown in Figure 3, point *A* represents the FWDDR’s rear wheel center, whereas point *B* denotes the reference trajectory’s preview point, distanced $l_{d}$ from point *A*. The robot takes point *O* as the circle center and *R* as the rotation radius. Following a circular motion with an angle of 2α, the robot reaches the preview point *B* to achieve the desired trajectory tracking.

To ensure that the rear wheel’s center point (point *A*) of the robot follows the dotted arc to reach point *B*, the following conditions must be satisfied:

(8) $$\begin{array}{cc} ∠AOB & =\pi -2∠CAB=\pi -2\left(\frac{\pi }{2}-\alpha \right)=2\alpha \end{array}$$

Note that in ▵AOB, the law of sine is satisfied and is given as:

(9) $$\frac{AB}{\sin(2\alpha )}=\frac{AO}{\sin(\frac{\pi }{2}-\alpha )}$$

Which can then be written as:

(10) $$\frac{l_{d}}{\sin(2\alpha )}=\frac{R}{\sin(\frac{\pi }{2}-\alpha )}$$

(11) $$\frac{l_{d}}{2\sin\alpha \cos\alpha }=\frac{R}{\cos\alpha }$$

The formula of robot radius of rotation can be simplified as:

(12) $$R=\frac{1}{2}\frac{l_{d}}{\sin\alpha }$$

Substituting (5) into (12), it can be obtained:

(13) $$\frac{l_{d}}{\sin\alpha }=\frac{b(v_{r}+v_{l})}{v_{r}-v_{l}}$$

By solving simultaneous Equations (4) and (13), the left and right wheel velocities of the FWDDR under PP algorithm can be obtained:

(14) $$\left\{\begin{array}{c} \frac{v_{r}}{v}=1+\frac{L\sin\alpha }{l_{d}}\\ \frac{v_{l}}{v}=1-\frac{L\sin\alpha }{l_{d}} \end{array}\right.$$

The lateral error between the mobile robot’s current pose and the target point, denoted as $e_{r}$, can be formulated as:

(15) $$e_{r}=l_{d}\sin\alpha$$

The lateral error $e_{r}$ can also be expressed as:

(16) $$e_{r}=\frac{l_{d}^{2}}{2L}\beta$$

where *L* is the long wheelbase of the FWDDR, β is the steering angle.

The equations highlight that the PP algorithm essentially acts as a *P* controller for lateral cornering, with the preview distance $l_{d}$ being crucial for effective tracking. In general, a linear relationship is established between the preview distance and the current vehicle speed, as defined in [32]:

(17) $$l_{d}=kv+l_{d0}$$

where *k* is an adjustable parameter, $l_{d0}$ is the preset preview distance, and *v* is the current vehicle speed.

Substituting (17) into (14), it can be obtained:

(18) $$\left\{\begin{array}{c} \frac{v_{r}}{v}=1+\frac{L\sin\alpha }{kv+l_{d0}}\\ \frac{v_{l}}{v}=1-\frac{L\sin\alpha }{kv+l_{d0}} \end{array}\right.$$

**Figure 4:**
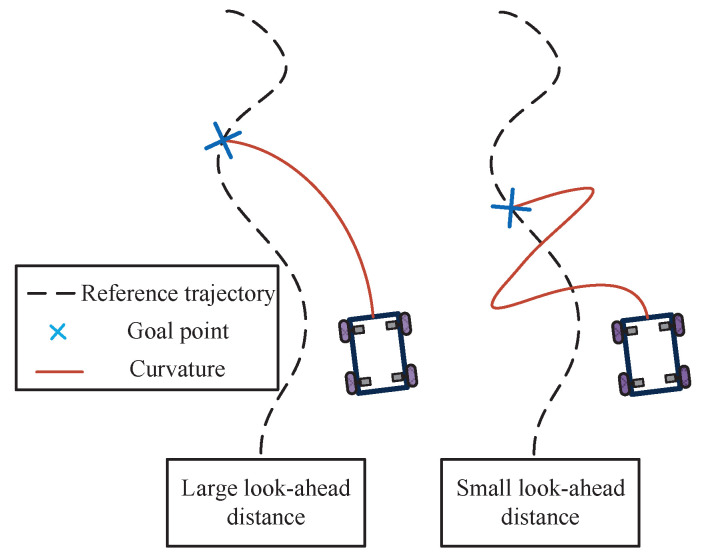
Renderings at different look-ahead distances.

The aforementioned formulas elucidate the relationship between *k* and the speeds of the left and right wheels. In fact, the preview distance profoundly influences control effects. When the look-ahead distance is longer, the control effect will be smoother, but it will lead to premature steering. Conversely, when the look-ahead distance is shorter, the control effect will be more accurate, but it will bring some oscillation and lead to an unstable tracking state, as shown in Figure 4.

### 3.2. Quadratic Polynomial-Based A-PP Control

As analyzed in the previous section, the look-ahead distance not only has a significant impact on the tracking effect but also affects the operational stability of the mobile robot to a certain extent. However, in a traditional PP algorithm, the look-ahead distance remains a constant value, which lacks the ability of randomization. Based on human driving habits, the look-ahead distance should correlate with parameters such as speed and path conditions.

Reference [33] considers the mathematical relationship between the look-ahead distance and the speed, and after several debug sessions, a formula was obtained for the relationship between the look-ahead distance and the speed:

(19) $$l_{d}=\frac{1}{6}v_{c}^{2}+\frac{1}{5}v_{c}+5$$

where $v_{c}$ is the speed of motion of the car.

In (19), only the vehicle’s speed influence on the look-ahead distance is considered, neglecting continuous speed changes and road curvature effects. Therefore, reference [34] introduced the consideration of path curvature and proposed a new look-ahead distance calculation method:

(20) $$l_{d}=k_{1}v+k_{2}\Omega +l_{d0}$$

where $k_{1}$ is the speed coefficient with positive value, $k_{2}$ is the road curvature coefficient with negative value, Ω is the road curvature, and $l_{d0}$ is the initial value of forward-looking distance.

To determine the optimal values, it is necessary to adjust the parameters $k_{1}$ and $k_{2}$. Equation (20) is usually applied to path tracking under constant speed conditions and may not be suitable for scenarios involving continuous speed changes. Therefore, to ensure stability during mobile robot navigation on curved roads and to maintain consistent speed changes, we propose a new A-PP control rooted in quadratic polynomials. When the mobile robot deviates from the desired path in terms of lateral position, the forward-looking distance is promptly adjusted and optimized to minimize lateral position errors. This enhances the effectiveness of path-tracking control and enables adaptive control, as outlined below:

(21) $$l_{d}=k_{1}v^{2}+k_{2}\varphi +k_{3}e_{r}+l_{d0}$$

where *v* is the linear velocity of the FWDDR and φ is the governing factor, $k_{1}$ can be expressed as:

(22) $$k_{1}=\frac{1}{2a_{\max}}$$

**Figure 5:**
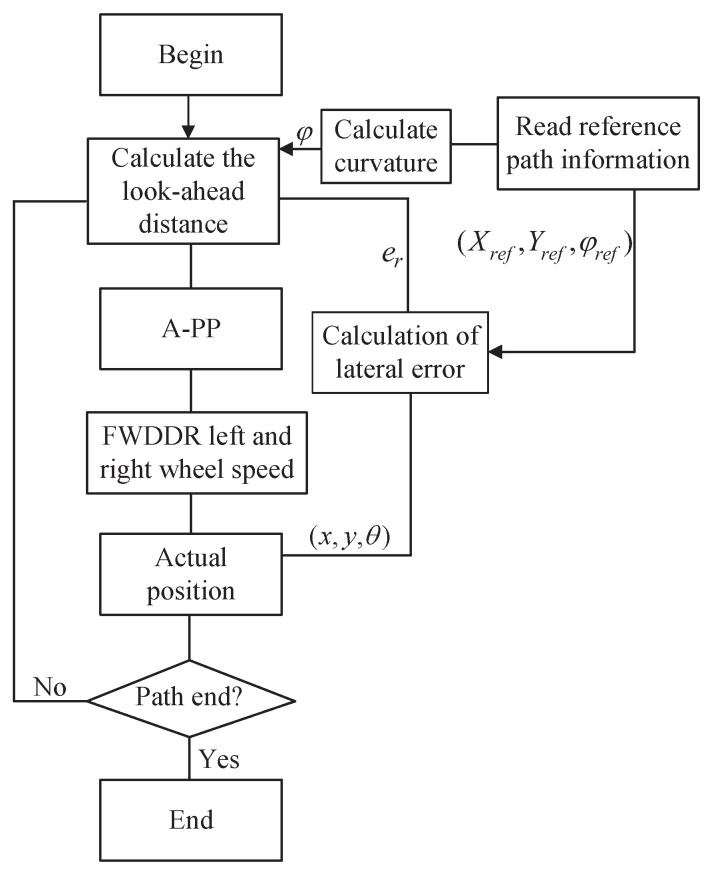
A-PP algorithm path-tracking flowchart.

Taking into account the robot’s nonholonomic constraints, the following parameters are relevant: $a_{max}$ represents the maximum acceleration of the FWDDR, $k_{2}$ is a real-time path curvature adjustment factor, $k_{3}$ is a real-time lateral error adjustment factor, and $l_{d0}$ is the initial forward-looking distance. Optimal values for $k_{2}$ and $k_{3}$ need to be determined through meticulous debugging. The flowchart of the A-PP algorithm is shown in Figure 5, and the specific steps are as follows:

Step 1: Retrieve reference path information, including horizontal and vertical coordinates $\left(X_{ref},Y_{ref}\right)$ and heading angle $\varphi_{ref}$.

Step 2: Calculate the path curvature and lateral error at each position, and use them as inputs in the forward-looking distance calculation formula. This allows for real-time determination of the appropriate forward-looking distance at the current position.

Step 3: Analyze the reference path by taking into account the look-ahead distance and FWDDR’s current position to identify the optimal preview point.

Step 4: Calculate the turning radius of the FWDDR using the PP algorithm’s principles. Convert the coordinates of the lookahead point to the robot’s coordinate system. This is achieved by translating the lookahead point’s coordinates to a system with the vehicle’s coordinates as the origin and then rotating this translated system by the heading angle of the differential drive robot to establish the final FWDDR coordinate system.

Step 5: Determine the FWDDR’s current position using its linear and angular velocities. Assess if it is the path’s endpoint. If so, conclude the program; otherwise, recalculate the preview distance.

In the lateral control of the A-PP algorithm, the error is multiplied with the adjustment factor and fed back to (21). When the error increases or decreases, the forward-looking distance is adjusted quickly and adaptively to eliminate the error as soon as possible and improve the response speed. In the longitudinal control, the reference path curvature and the adjustment factor are multiplied and fed back to (21) to adaptively adjust the forward-looking distance online, especially in the turning road condition.

### 3.3. Road Curvature Calculation Method

Given three consecutive discrete points (*A*, *B*, *C*), the parametric curve equation of three adjacent discrete points is as follows:

(23) $$\left\{\begin{array}{c} x\left(t\right)=a_{0}+a_{1}t+a_{2}t^{2}\\ y\left(t\right)=b_{0}+b_{1}t+b_{2}t^{2} \end{array}\right.$$

where $a_{0}$, $a_{1}$, $a_{2}$ and $b_{0}$, $b_{1}$, $b_{2}$ are the undetermined coefficients.

**Figure 6:**
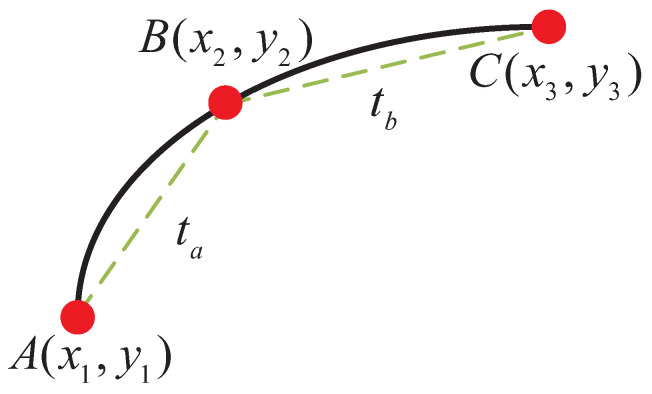
Three-point parametric equation.

The three-point parametric equation diagram is shown in Figure 6. Additionally, the curvature calculation formula for a parameter curve is as follows:

(24) $$k=\frac{x^{\prime \prime }y^{\prime }-x^{\prime }y^{\prime \prime }}{{\left(x^{\prime 2}+y^{\prime 2}\right)}^{3/2}}=\frac{a_{2}b_{1}-a_{1}b_{2}}{{\left(a_{1}^{2}+b_{1}^{2}\right)}^{3/2}}$$

Obviously, to accurately calculate the curvature of the parameter curve in (24), it is essential to first determine the six undetermined coefficients in Equation (23). The lengths of the two vectors derived from the three discrete points can be denoted as:

(25) $$\left\{\begin{array}{c} t_{a}=\sqrt{{\left(x_{2}-x_{1}\right)}^{2}+{\left(y_{2}-y_{1}\right)}^{2}}\\ t_{b}=\sqrt{{\left(x_{3}-x_{2}\right)}^{2}+{\left(y_{3}-y_{2}\right)}^{2}} \end{array}\right.$$

where $t_{a}$ is the distance between discrete points A and B, and $t_{b}$ is the distance between discrete points B and C.

Given the proximity of points *A*, *B*, and *C*, the central angle of the corresponding curvature radius remains relatively consistent. Consequently, we can approximate the arc AB length to the straight-line distance between *A* and *B*. For Equation (23), this implies that the variation in the independent variable *t* adheres to the approximation:

(26) $$\left\{\begin{array}{c} \left(x,y\right){|}_{t=-t_{a}}=\left(x_{1},y_{1}\right)\\ \left(x,y\right){|}_{t=0}=\left(x_{2},y_{2}\right)\\ \left(x,y\right){|}_{t=t_{b}}=\left(x_{3},y_{3}\right) \end{array}\right.$$

By substituting (26) into (25), the following can be obtained:

(27) $$\left\{\begin{array}{c} x_{1}=a_{0}-a_{1}t_{a}+a_{2}t_{a}^{2}\\ x_{2}=a_{0}\\ x_{3}=a_{0}+a_{1}t_{b}+a_{2}t_{b}^{2} \end{array}\right.,\left\{\begin{array}{c} y_{1}=b_{0}-b_{1}t_{a}+b_{2}t_{a}^{2}\\ y_{2}=b_{0}\\ y_{3}=b_{0}+b_{1}t_{b}+b_{2}t_{b}^{2} \end{array}\right.$$

The preceding formula can be reformulated in matrix representation:

(28) $$\left\{\begin{array}{c} \left[\begin{array}{c} x_{1}\\ x_{2}\\ x_{3} \end{array}\right]=\left[\begin{array}{ccc} 1 & -t_{a} & t_{a}^{2}\\ 1 & 0 & 0\\ 1 & t_{b} & t_{b}^{2} \end{array}\right]\left[\begin{array}{c} a_{0}\\ a_{1}\\ a_{2} \end{array}\right]\\ \left[\begin{array}{c} y_{1}\\ y_{2}\\ y_{3} \end{array}\right]=\left[\begin{array}{ccc} 1 & -t_{a} & t_{a}^{2}\\ 1 & 0 & 0\\ 1 & t_{b} & t_{b}^{2} \end{array}\right]\left[\begin{array}{c} b_{0}\\ b_{1}\\ b_{2} \end{array}\right] \end{array}\right.$$

Note:

(29) $$\begin{array}{c} X=\left[\begin{array}{c} x_{1}\\ x_{2}\\ x_{3} \end{array}\right],Y=\left[\begin{array}{c} y_{1}\\ y_{2}\\ y_{3} \end{array}\right]\\ A=\left[\begin{array}{c} a_{0}\\ a_{1}\\ a_{2} \end{array}\right],B=\left[\begin{array}{c} b_{0}\\ b_{1}\\ b_{2} \end{array}\right]\\ M=\left[\begin{array}{ccc} 1 & -t_{a} & t_{a}^{2}\\ 1 & 0 & 0\\ 1 & t_{b} & t_{b}^{2} \end{array}\right] \end{array}$$

Subsequently, the matrix of undetermined coefficients can be written as:

(30) $$\left\{\begin{array}{c} A=M^{-1}X\\ B=M^{-1}Y \end{array}\right.$$

where $M^{-1}$ is the inverse matrix of M.

Finally, by substituting (30) into (24), the curvature can be obtained.

## 4. Path Planning and Tracking

**Figure 7:**
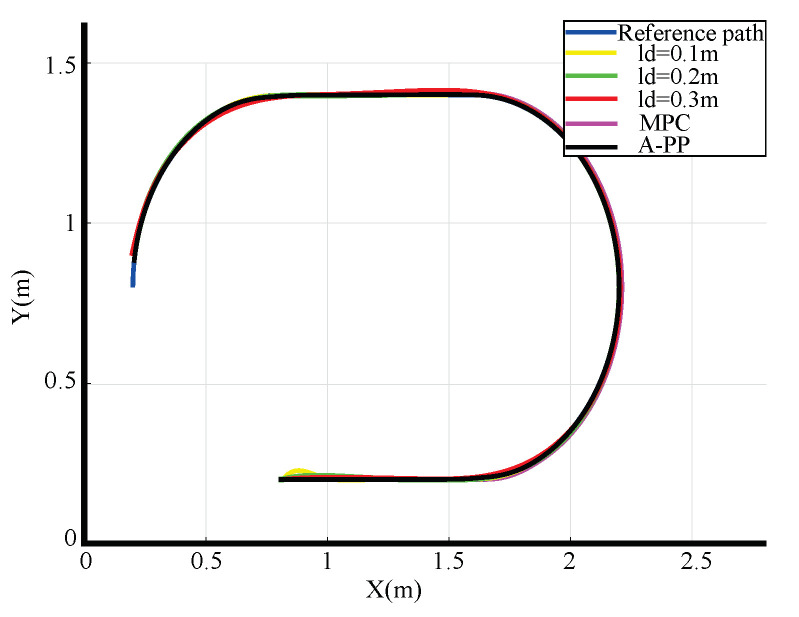
Simulation path-tracking effect.

In this design, the stability of the mobile robot when tracking both straight and curved paths will be verified. The coordinates of the starting and ending points are (0.8, 0.2) and (0.2, 0.6), respectively. The reference path is shown in the blue curve in Figure 7. In this comparative simulation, the feasibility of the A-PP algorithm is assessed. The mobile robot’s speed is fixed at 0.2 m/s, whereas the look-ahead distance varies (0.1 m in yellow, 0.2 m in green, and 0.3 m in red) for comparison with the proposed A-PP algorithm. At the same time, compared with the current mainstream MPC algorithm, the adaptability of A-PP algorithm is evaluated.

**Figure 8:**
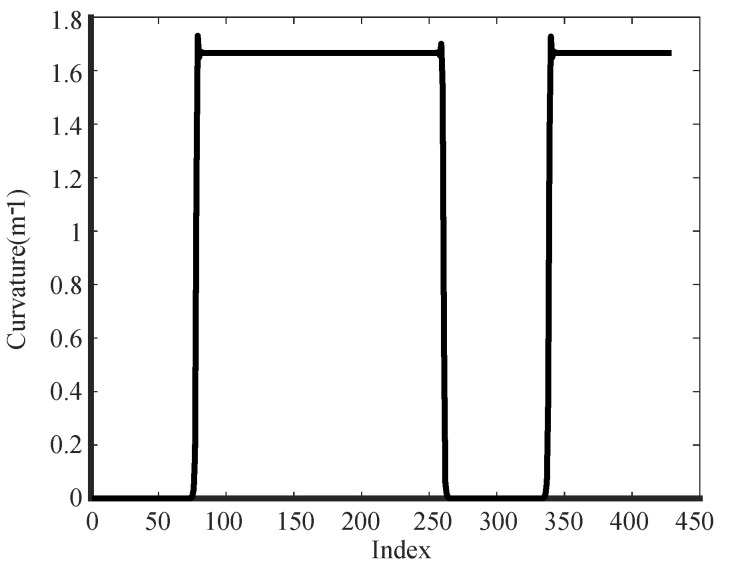
Simulation road curvature.

Using the three-point parametric equation method (as per (24)), we derive the road curvature depicted in Figure 8. It can be seen that the curved part of the reference path exhibits a maximum curvature value of 1.66679. Conversely, the straight part holds a minimum curvature value close to 0.

**Figure 9:**
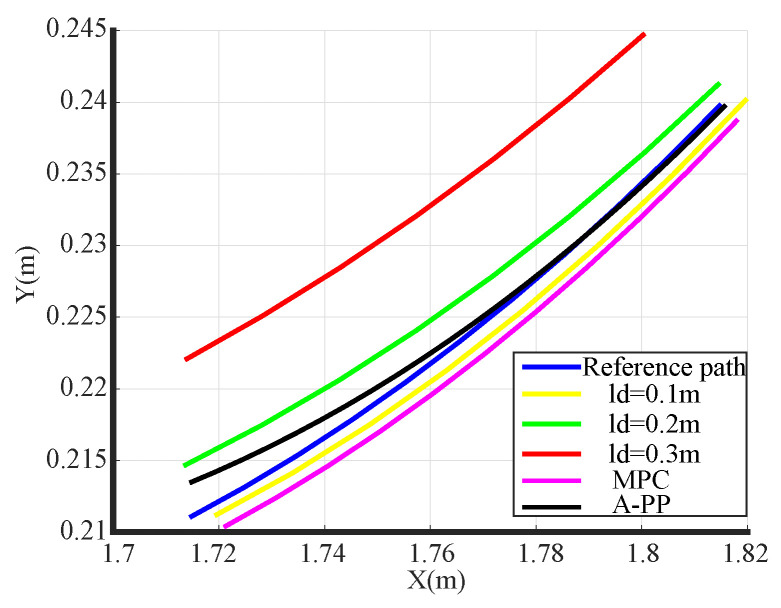
Simulation of partial curve enlargement.

**Figure 10:**
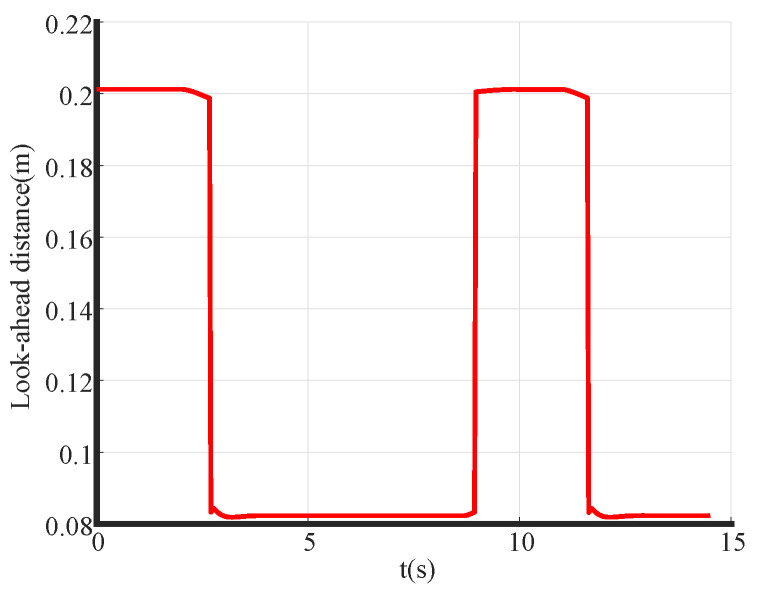
Simulation forward view distance.

The simulation tracking results are shown in Figure 7. With a look-ahead distance of 0.1 m (yellow), significant control oscillations are observed, especially during the initial phase, resulting in considerable tracking errors and poor alignment with the reference path. In contrast, with the A-PP algorithm, the robot starts without pronounced oscillations and maintains stability throughout. The simulation of partial curve enlargement is shown in Figure 9. It is evident that a significant curvature change occurs when entering the curve. At this time, the A-PP algorithm can rapidly make necessary adjustments, enabling it to closely follow the desired path and enhancing the stability and reliability of the mobile robot. However, with forward-looking distances of 0.2 m (green) and 0.3 m (red), significant lateral errors occur on curved roads. This inconsistency arises because traditional algorithms use a fixed forward-looking distance that remains unaltered, irrespective of road conditions. MPC has improved stability compared to the traditional PP algorithm, but it is not as effective as the A-PP algorithm in terms of tracking effectiveness, especially in road conditions with large curvature changes. In Figure 10, it is evident that as the mobile robot travels in a straight line, the curvature decreases, whereas the forward distance increases. Conversely, as the robot reaches a turning position, the curvature increases, and the forward distance decreases. Forward distance adjusts according to the reference path’s curvature, ranging between 0.08 m and 0.2 m.

**Figure 11:**
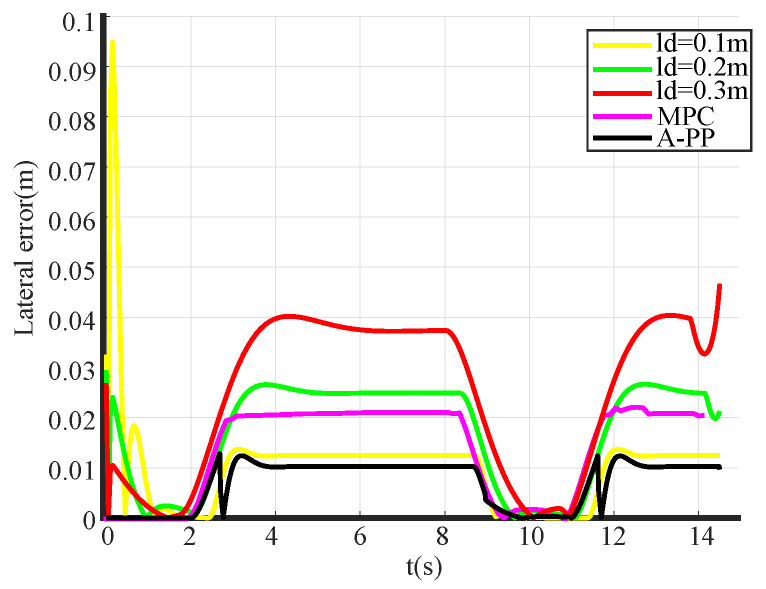
Simulation lateral error of FWDDR.

The simulation’s lateral error diagram is shown in Figure 11. It is evident that, compared to traditional PP algorithms, the A-PP algorithm exhibits negligible lateral errors on straight paths. Upon entering a curved road, lateral errors increase; however, the A-PP algorithm promptly adjusts to maintain the mobile robot’s stable operation with minimal error. The MPC algorithm has a similar tracking effect compared to A-PP in straight-line road conditions, but when encountering road conditions with large changes in curvature, the tracking error of MPC increases, whereas the A-PP has a smaller tracking error and is able to respond quickly according to the road conditions. As illustrated in Table 1, while tracking the desired path, the A-PP algorithm achieves an average lateral error of 0.00694 m, a variance of 0.004663 m, and a maximum lateral error of 0.012837 m. The stability and accuracy are notably improved when compared to the traditional PP and MPC algorithms.

## 5. Experiment

To further validate the practicality of the A-PP algorithm, experimental verification is conducted using a physical FWDDR as the platform.

### 5.1. Experimental Setup

The overall design specifications of the FWDDR are shown in Table 2:

The following describes the workflow of the four-wheel differential electric drive system:

(1) Control commands are sent through the PC Matlab/Simulink control interface via the RS485 bus;
(2) On the PC side, E32-DTU converts commands transmitted via the RS485 bus into LoRa RF signals;
(3) On the FWDDR side, E32-DTU converts LoRa signals into RS485 signals;
(4) The motion controller interprets RS485 signals based on the four-wheel differential model and generates corresponding servo drive commands. These commands are then transmitted to the four servo drives through the CAN bus;
(5) The motion controller transmits motor status, battery voltage, and other parameters back to the upper computer through a reverse path.

### 5.2. Experiment Process

**Figure 12:**
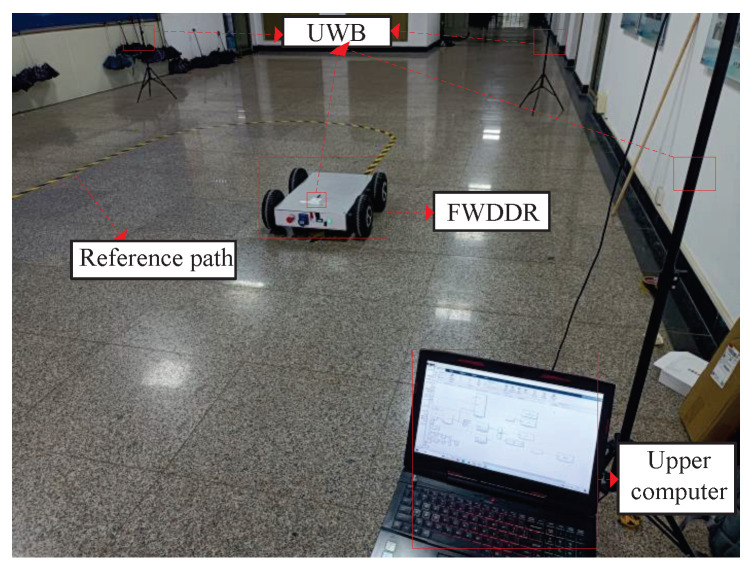
Experimental site.

The experimental site is shown in Figure 12, where location is obtained through a UWB device based on the DW1000 protocol. The maximum distance measurement range is 150 m, with positioning accuracy reaching the centimeter level. A rectangular positioning area is established using four base stations, with the reference trajectory positioned within this area. One of the base stations is connected to the upper computer via a cable. Furthermore, a UWB tag is positioned at the rear wheel’s center on the FWDDR. As the FWDDR moves, the tag continuously updates its coordinate position to record the FWDDR’s motion trajectory.

In this experiment, the A-PP algorithm was compared and analyzed with traditional PP and MPC algorithms to validate A-PP’s superiority. We set the speed at 0.1 m/s and used a forward-looking distance of 0.2 m for the experiment. After multiple rounds of parameter-tuning tests, the tracking effect is best when $k_{1}=\frac{1}{4}$, $k_{2}=-0.07$, $k_{3}=-0.2$.

### 5.3. Analysis of Experimental Results

**Figure 13:**
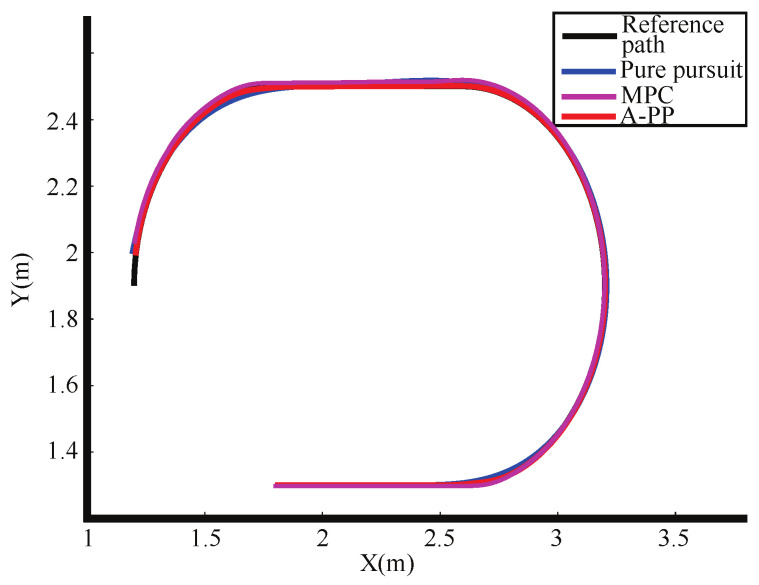
Experimental tracking effect.

**Figure 14:**
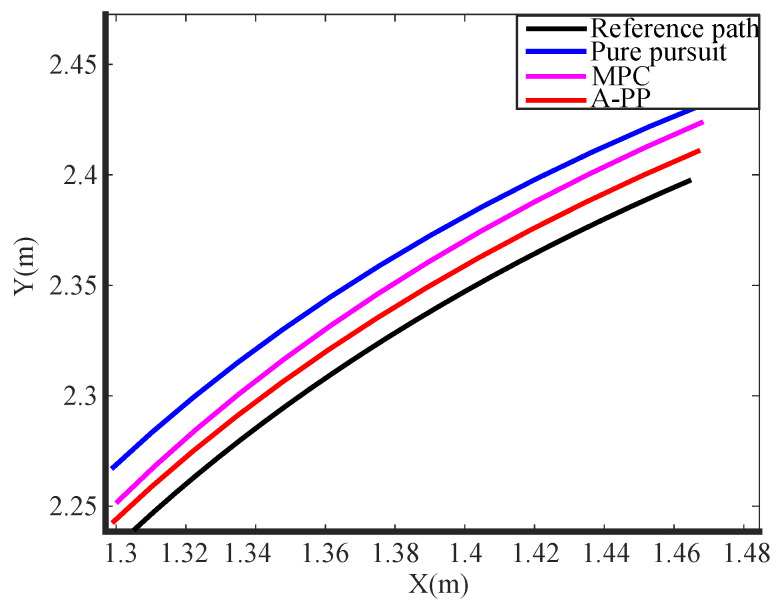
Enlarged view of the experimental partial bend.

**Figure 15:**
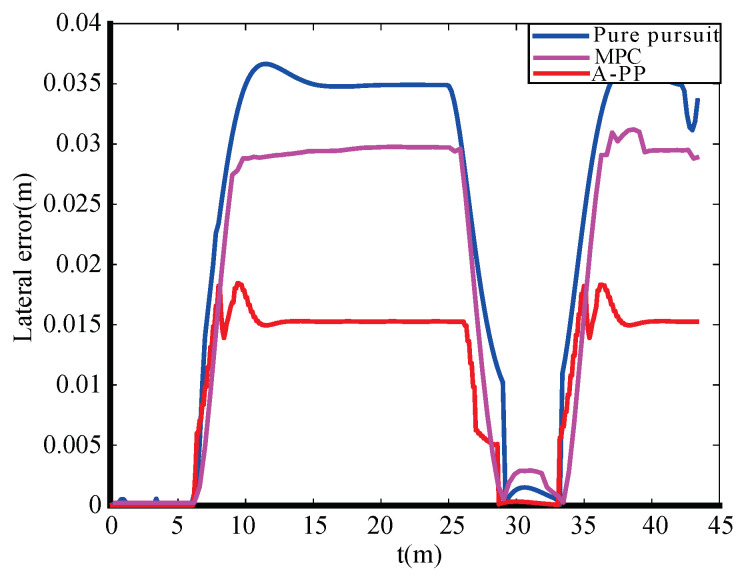
Experimental lateral error.

The experimental tracking results, along with an enlarged view of a partial bend in the experiment, are depicted in Figure 13 and Figure 14. An observation from the figures reveals that the traditional PP and MPC algorithms track the desired path well under straight road conditions. However, it exhibits significant tracking errors when encountering curved-road conditions, failing to meet the design requirements. In contrast, the A-PP algorithm achieves adaptive adjustments by controlling in both the lateral and longitudinal directions. This enables it to better track both straight-line and curved-road conditions to meet the design requirements. As evident from the experimental lateral error in Figure 15, the traditional PP algorithm exhibits slight oscillations at startup, whereas the A-PP algorithm operates stably with high tracking accuracy on straight sections. When the road surface curvature undergoes significant changes upon entering a curve, A-PP can swiftly make necessary adjustments. In contrast, the traditional pp and MPC algorithms have large errors on the curve and cannot achieve accurate tracking. The experiment error comparison table is presented in Table 3. When the A-PP algorithm tracks the desired path, the average lateral error measures 0.01070 m, with a variance of 0.006663 m and a maximum lateral error of 0.019443 m. These values represent a significant enhancement in stability and accuracy compared to the traditional PP and MPC algorithms.

## 6. Conclusions

In the field of mobile robot path tracking, achieving a precise trajectory following is of utmost importance. However, traditional methods often lack real-time adjustment capability for the forward-looking distance. In this study, we proposed and verified the A-PP algorithm, which incorporates a quadratic polynomial in both lateral and longitudinal dimensions. The main findings of the paper are as follows:

(1) The quadratic polynomial is enhanced in both lateral and longitudinal dimensions to facilitate adaptive dynamic adjustment of the forward-looking distance. This enhancement reduces the lateral deviation of the FWDDR during path tracking and enhances both tracking accuracy and operational stability.
(2) The A-PP algorithm is simulated and verified by Matlab/Simulink, and the results indicate that the A-PP algorithm achieves a mean lateral error of 0.00694 m, a variance of 0.004663 m, and a maximum lateral error of 0.012837 m during path tracking, which represent a significant enhancement in stability and accuracy when compared to the traditional PP and MPC algorithms.
(3) Experimental tests further validated the A-PP algorithm. The results showed a mean lateral error of 0.01070 m, a variance of 0.006663 m, and a maximum lateral error of 0.019443 m. In comparison with the PP algorithm, the A-PP algorithm achieved faster convergence of deviations, enhanced lateral errors in turning sections, and heightened driving smoothness, all while maintaining low computational time.

This research shows that the A-PP algorithm has the advantages of adaptive control, accurate tracking, fast computation speed, and excellent robustness. However, there are some limitations, such as inaccurate tracking under high-speed motion and large localization deviation of UWB, etc. In future work, further improvement and optimization will be conducted to address these limitations. As road conditions become increasingly complex, the exploration and further refinement of such algorithms will be instrumental in ensuring safe and precise vehicle operations in the future.

## Figures and Tables

**Table 1:** Simulation error comparison table.

Method	Average Value of Lateral Error (m)	Variance (m)	Maximum Lateral Error (m)
$L_{d}$ = 0.1 m	0.01007	0.010075	0.094842
$L_{d}$ = 0.2 m	0.01688	0.010333	0.029417
$L_{d}$ = 0.3 m	0.02462	0.015404	0.046663
MPC	0.01467	0.010247	0.021854
A-PP	0.00694	0.004663	0.012837

**Table 2:** Overall design specifications.

Parameters	Value	Units
Length	770	mm
Width	658	mm
Wheelbase	470	mm
Wheel tread	573	mm
Minimum turning	1015	mm
Tire size (diameter)	260	mm
Maximum motor speed	3600	rpm
Maximum driving torque	47.5	N·m
Maximum load	≥50	kg

**Table 3:** Experiment error comparison table.

Method	Average Value of Lateral Error (m)	Variance (m)	Maximum Lateral Error (m)
Pure pursuit	0.02372	0.016588	0.036705
MPC	0.02069	0.014416	0.031628
A-PP	0.01070	0.006663	0.019443

## Data Availability

The data generated and/or analyzed as well as the source code used in the current study are not publicly available due to their use in an ongoing project, but may be available from the corresponding author on reasonable request.
